# TRIB3 silencing promotes the downregulation of Akt pathway and PAX3-FOXO1 in high-risk rhabdomyosarcoma

**DOI:** 10.1186/s40164-024-00503-9

**Published:** 2024-04-05

**Authors:** Gabriel Gallo-Oller, Guillem Pons, Júlia Sansa-Girona, Natalia Navarro, Patricia Zarzosa, Lia García-Gilabert, Paula Cabré-Fernandez, Gabriela Guillén Burrieza, Lorena Valero-Arrese, Miguel F. Segura, José M. Lizcano, José Sánchez de Toledo, Lucas Moreno, Soledad Gallego, Josep Roma

**Affiliations:** 1 Childhood Cancer and Blood Disorders, Vall d’Hebron Research Institute (VHIR), Barcelona, Spain; 2 Paediatric Surgery Department, Vall d’Hebron University Hospital, Barcelona, Spain; 3 Paediatric Oncology and Haematology Department, Vall d’Hebron University Hospital, Barcelona, Spain; 4 Departament de Bioquímica i Biologia Molecular and Institut de Neurociències. Facultat de Medicina, Universitat Autònoma de Barcelona (UAB), Barcelona, Spain; 5 Protein Kinases in Cancer Research. Vall d’Hebron Institut de Recerca (VHIR), Barcelona, Spain; 6 Departament de Pediatria, Facultat de Medicina, Universitat Autònoma de Barcelona (UAB), Barcelona, Spain

**Keywords:** TRIB3, Fusion protein, PAX3-FOXO1, Akt, Rhabdomyosarcoma

## Abstract

Rhabdomyosarcoma (RMS), such as other childhood tumors, has witnessed treatment advancements in recent years. However, high-risk patients continue to face poor survival rates, often attributed to the presence of the PAX3/7-FOXO1 fusion proteins, which has been associated with metastasis and treatment resistance. Despite efforts to directly target these chimeric proteins, clinical success remains elusive. In this study, the main aim was to address this challenge by investigating regulators of FOXO1. Specifically, we focused on TRIB3, a potential regulator of the fusion protein in RMS. Our findings revealed a prominent TRIB3 expression in RMS tumors, highlighting its correlation with the presence of fusion protein. By conducting TRIB3 genetic inhibition experiments, we observed an impairment on cell proliferation. Notably, the knockdown of TRIB3 led to a decrease in PAX3-FOXO1 and its target genes at protein level, accompanied by a reduction in the activity of the Akt signaling pathway. Additionally, inducible silencing of TRIB3 significantly delayed tumor growth and improved overall survival in vivo. Based on our analysis, we propose that TRIB3 holds therapeutic potential for treating the most aggressive subtype of RMS. The findings herein reported contribute to our understanding of the underlying molecular mechanisms driving RMS progression and provide novel insights into the potential use of TRIB3 as a therapeutic intervention for high-risk RMS patients.

**Supplementary Information:**

The online version contains supplementary material available at 10.1186/s40164-024-00503-9.

## To the editor,

Rhabdomyosarcoma (RMS) stands as the most prevalent soft tissue malignant tumor in childhood, encompassing 4–5% of all childhood cancers [1]. Over the past few decades, treatment advancements have improved outcomes for RMS patients. However, patients classified as high-risk still exhibit a survival rate of only 40% [2, 3]. In recent years, there has been a notable shift in focus towards molecular markers in the risk stratification of RMS. Specifically, two genotypes have been identified: (1) those with *PAX3-FOXO1* or *PAX7-FOXO1* gene fusions (fusion positive: FP-RMS); and (2) those without these fusions (fusion negative: FN-RMS), with FP-RMS tumors exhibiting the highest rates of metastatic progression and therapy failure [4, 5]. Given their crucial role in the etiology of the disease and their specificity, fusion proteins are regarded as promising molecular targets for RMS. Consequently, numerous studies have focused on elucidating their molecular regulation to develop inhibitory approaches for therapeutic purposes [4, 6]. Within this context, a FOXO1 key regulator is the tribbles homolog 3 protein (TRIB3), which plays a pivotal role in diverse malignancies [7, 8]. TRIB3 belongs to a subfamily of pseudokinases comprising three members: TRIB1, TRIB2, and TRIB3 that share a highly conserved, non-functional kinase-like domain [9]. While the role of TRIB3 has been studied in several tumor types, its involvement in RMS remains unexplored. Of note, a previous study by our team [10] pointed TRIB3 as a possible regulator of chemoresistance in RMS, suggesting that TRIB3 may play significant functional roles in this tumor.

**Fig. 1:**
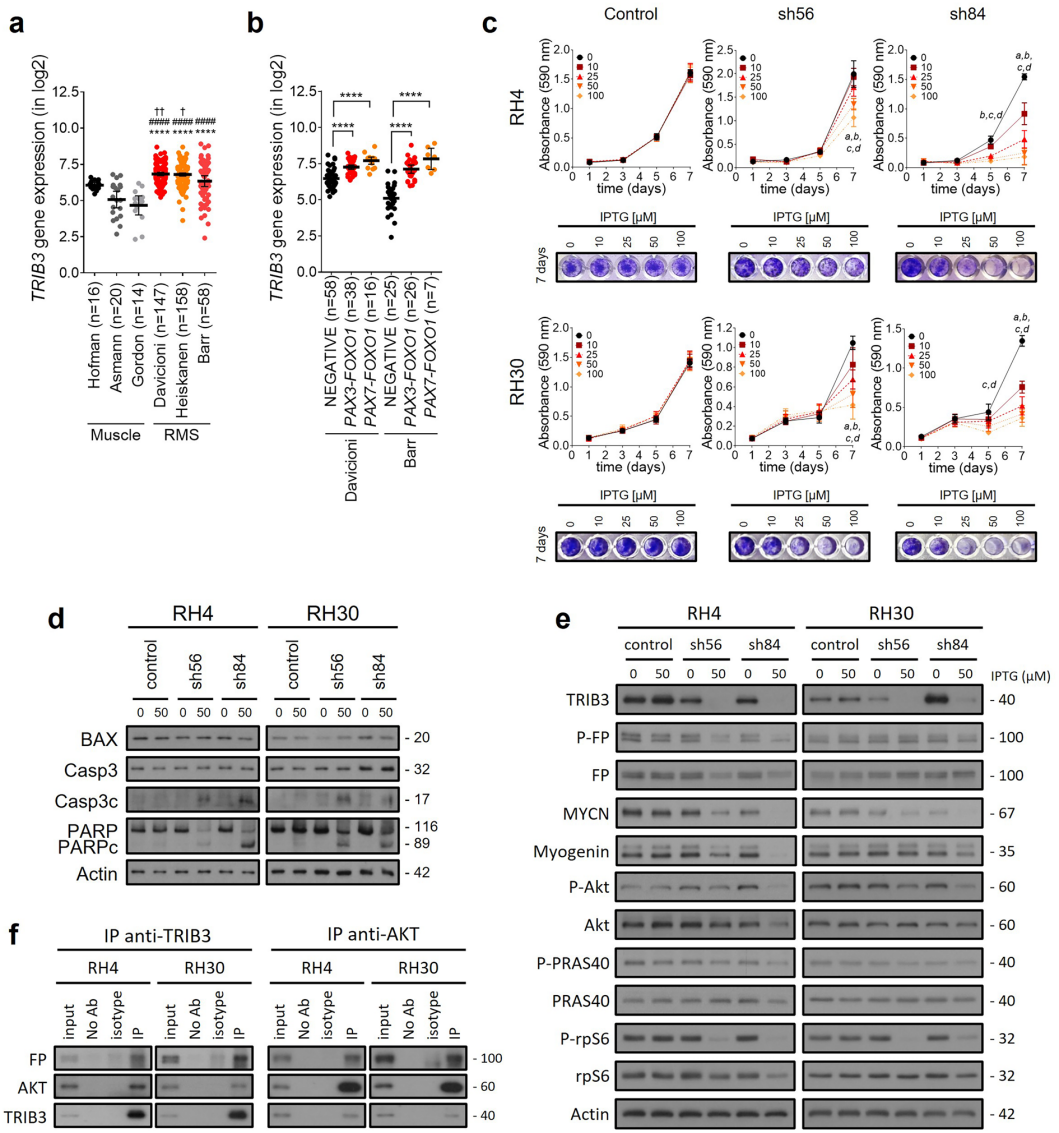
Analysis of TRIB3's role in RMS. Expression data obtained from patients' datasets on the R2 platform were used. The data are presented as the mean ± 95% confidence interval. **a** TRIB3 was found to be overexpressed in RMS datasets compared to muscle healthy counterpart. One-way ANOVA was performed, and Tukey's multiple comparisons test was used as the post hoc test. Significance versus Hofman is indicated by †, versus Asmann by #, and versus Gordon by*. **b** Tumors harboring PAX3-FOXO1 or PAX7-FOXO1 fusion gene displayed higher TRIB3 expression levels. One-way ANOVA with Dunnett's post hoc test was used to determine significance. **c** Proliferation was assessed using the inducible model and the crystal violet assay at different IPTG concentrations. A two-way ANOVA followed by Dunnett's multiple comparisons test was performed to compare each IPTG concentration with the non-induced condition at each time point. **d** Apoptosis was analyzed by determining apoptotic markers through WB. **e** Analysis of fusion protein and its phosphorylation status by WB, along with MYCN and myogenin proteins as fusion protein target genes, confirmed that PAX3-FOXO1 function is affected after TRIB3 knockdown. Akt signaling pathway inhibition after TRIB3 knockdown was also analyzed by WB. Phosphorylation status of Akt and its main downstream effectors (PRAS40 and rpS6) was assessed. **f** Immunoprecipitations immobilizing TRIB3 (left panel) or Akt (right panel were carried out using cell lysates from RH4 and RH30 cells. Negative control was included (beads only and no antibody), and isotype-matched IgG was used as a control. FP: fusion protein. P: phosphorylated. IP: immunoprecipitation

In this work, we present the first functional characterization of the role of TRIB3 in RMS, particularly its association with PAX3-FOXO1 and its potential as a therapeutic target. Our study reveals that TRIB3 is overexpressed in RMS (Fig. 1a), especially in tumors (Fig. 1b) and cell lines (Additional file 1: Fig. S1) carrying PAX3-FOXO1 or PAX7-FOXO1 fusions. Functional characterization of TRIB3 in RMS was achieved through silencing experiments using constitutive (Additional file 1: Fig. S1) and inducible models (Fig. 1). TRIB3 silencing resulted in impaired proliferation (Fig. 1c) and increased apoptosis (Fig. 1d), with higher sensitivity in FP-RMS cells. Moreover, our study demonstrated a reduction in PAX3-FOXO1 levels following TRIB3 knockdown in RH4 cells, together with a reduction in its target genes MYCN and myogenin, suggesting that TRIB3 may regulate the stability and activity of PAX3-FOXO1. Interestingly, gene expression levels of the fusion protein, remained unaltered after TRIB3 knockdown (Additional file 1: Fig. S2a). In addition, TRIB3 knockdown led to downregulation of Akt signaling, as evidenced by a reduction in the phosphorylation of Akt and its downstream effectors such as PRAS40 and rpS6 (Fig. 1e and Additional file 1: Fig. S2b for blot quantification). Importantly, our experimental findings reveal an interaction between TRIB3, PAX3-FOXO1, and Akt. Coimmunoprecipitation assays demonstrated the association of TRIB3 with both PAX3-FOXO1 and Akt, suggesting a potential role of TRIB3 in the regulatory mechanisms involving PAX3-FOXO1 in RMS. Notably, reciprocal coimmunoprecipitation experiments further confirmed the association of TRIB3 with PAX3-FOXO1 and Akt (Fig. 1f). These results suggest a dynamic interplay between TRIB3, PAX3-FOXO1, and Akt, shedding light on the intricate regulatory networks underlying RMS pathobiology. While TRIB3 and PAX3-FOXO1 interaction was previously proposed through in silico analysis [11], experimental confirmation was lacking.

**Fig. 2:**
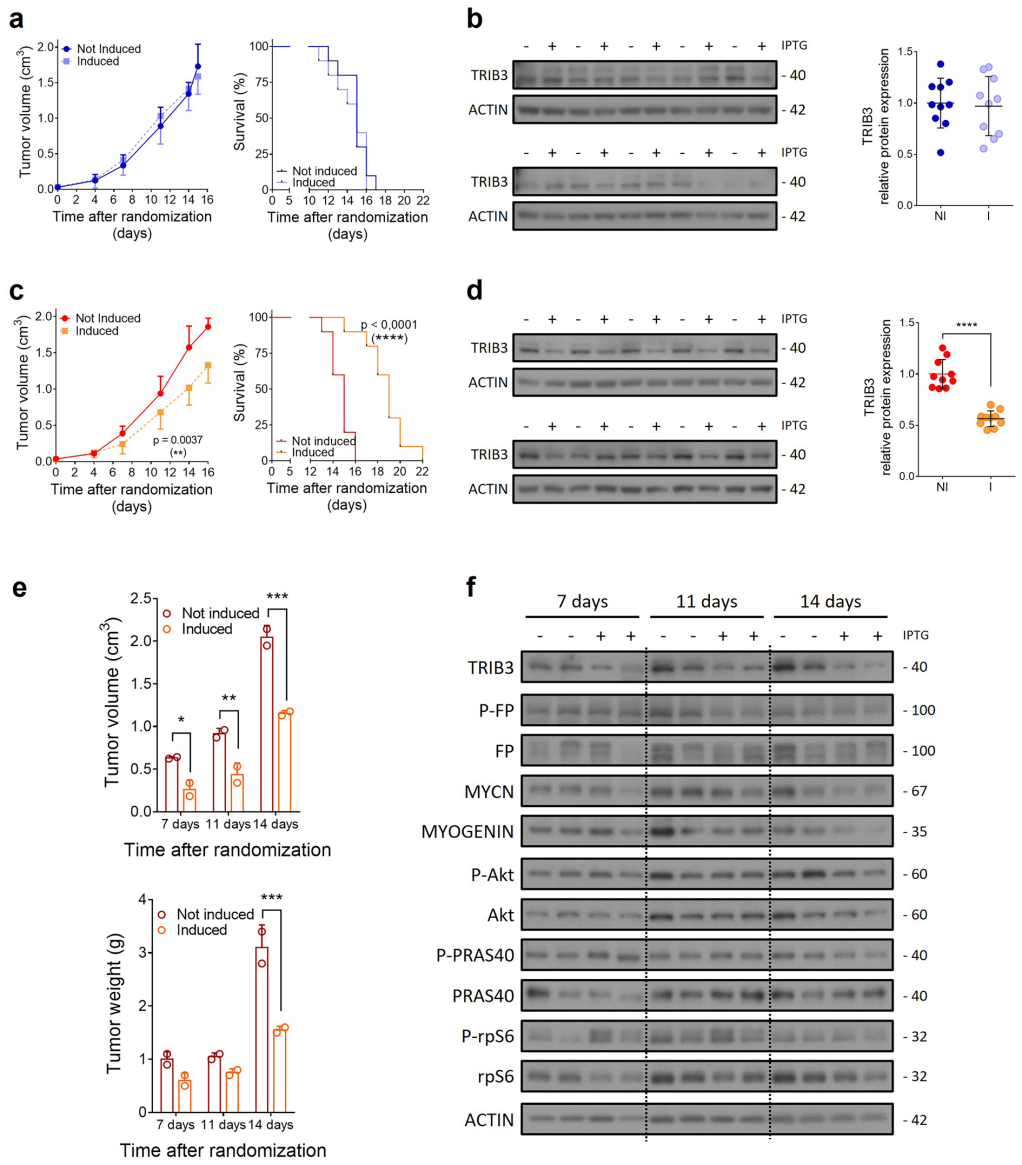
Silencing of TRIB3 inhibits in vivo tumor growth. An in vivo experiment was conducted using the inducible pLKO-3xLacO system in the RH30 cell line. **a** Tumor growth (left panel) and survival (right panel) of control plasmid-derived tumors showed no significant differences after induction. **b** TRIB3 protein levels in tumors were analyzed by WB (left panel) and quantified (right panel). **c** Induction of sh84 in tumor cells resulted in delayed tumor growth (left panel) and improved survival (right panel). **d** WB analysis revealed decreased TRIB3 protein levels after IPTG induction (left panel), with a significant reduction of approximately 50% observed in induced tumors at the end of the experiment (right panel). **e** Mice from the sh84 group, sacrificed at 7, 11, and 14 days, showed a significant decrease in both tumor volume (upper panel) and tumor weight (lower panel). **f** Analysis of tumor samples by WB showed a decrease in MYCN and myogenin levels, along with decreases in total Akt levels and in the phosphorylation status of its substrate PRAS40. In (**b**, **d**) each lane represents a sample from an individual mouse. FP: fusion protein. P: phosphorylated

Remarkably, in vivo experiments (Fig. 2) showed that the TRIB3 silencing produced a delay in tumor growth and improved overall survival (Fig. 2a, c). At the end of the experiment, the TRIB3-silenced group exhibited a notable reduction of approximately 50% in TRIB3 levels (Additional file 1: Fig. 2b, d). At 7-, 11- and 14-days post-induction of TRIB3 silencing, a decrease in tumor volume and tumor weight was observed (Fig. 2e). Regarding the downstream effectors, a decrease in PAX3-FOXO1 target genes MYCN and myogenin was observed after 14 days of induction with IPTG (Fig. 2f and Additional file 1: Fig. S3b for blot quantification). Regarding Akt signaling pathway, only a decrease in total Akt levels was observed at 14 days, along with a decrease in PRAS40 phosphorylation at the same time point. These results validated the impact of TRIB3 genetic inhibition on RMS cell proliferation, offering insights into its role in modulating PAX3-FOXO1 target genes and Akt signaling in a physiological setting.

In conclusion, this study provides valuable insights into the crucial role of TRIB3 in RMS pathogenesis, particularly in PAX3-FOXO1 tumors, the most aggressive RMS subtype. The overexpression of TRIB3 in RMS, its functional impact on proliferation, apoptosis, PAX3-FOXO1 regulation, and Akt signaling support its potential as a therapeutic target. Our data reveal an interaction between TRIB3, Akt, and PAX3-FOXO1, as demonstrated by coimmunoprecipitation experiments. These results provide experimental evidence of the association among TRIB3, Akt, and PAX3-FOXO1 in our FP-RMS model. Although these data indicate physical interactions, they do not imply a direct regulatory relationship. While the regulatory axis of TRIB3/Akt/FOXO1 has been reported in other malignancies [7, 8, 12], our study specifically establishes the physical associations between TRIB3, Akt, and PAX3-FOXO1 in FP-RMS. Further investigations are warranted to elucidate the functional consequences of these interactions and to determine the specific regulatory mechanisms involved in modulating PAX3-FOXO1 expression in the context of TRIB3 and Akt.. These findings offer new avenues for therapeutic development and underscore the need for further research to validate TRIB3 as a promising candidate for targeted therapy in RMS. The knowledge gained from this study contributes to our understanding of the complex molecular landscape of RMS and facilitates the way for more effective treatments to improve patient outcomes.

### Supplementary Information

**Additional file 1.** Materials and Methods. **Table S1**: Western blot antibodies conditions. **Figure S1**: TRIB3 is overexpressed in FP-RMS cell lines, and its genetic inhibition leads to impaired cell survival and increased apoptosis. **Figure S2**: Gene expression levels determination and band intensity quantification of WB results from Figure 1e. **Figure S3**: Scheme of the in vivo experimental design and band intensity quantification for mice samples sacrificed at 7, 11, and 14 days.

## Data Availability

The datasets used and/or analyzed during the current study are available in the public R2 database repository: [http://r2.amc.nl]. All requests should be submitted to the corresponding author and are available upon reasonable request.
