# Supplementary material for: TRIB3 silencing promotes the downregulation of Akt pathway and PAX3-FOXO1 in high-risk rhabdomyosarcoma
Source: Exp Hematol Oncol. 2024 Apr 5;13:38. doi: 10.1186/s40164-024-00503-9 (PMC10996176; doi:10.1186/s40164-024-00503-9)
Supplement: Supplementary file 1 — Additional file 1. Materials and Methods. Table S1: Western blot antibodies conditions. Figure S1: TRIB3 is overexpressed in FP-RMS cell lines, and its genetic inhibition leads to impaired cell survival and increased apoptosis. Figure S2: Gene expression levels determination and band intensity quantification of WB results from Figure 1e. Figure S3: Scheme of the in vivo experimental design and band intensity quantification for mice samples sacrificed at 7, 11, and 14 days. [file 40164_2024_503_MOESM1_ESM.docx]

**TRIB3 silencing promotes the downregulation of Akt pathway and PAX3-FOXO1 in high-risk rhabdomyosarcoma**

Gallo-Oller et al.

**ADDITIONAL FILE**

**Material and methods**

*Mining transcriptome data for rhabdomyosarcoma*

We conducted an mRNA expression analysis using the R2 Genomics Analysis and Visualization platform (http://r2.amc.nl). The following datasets were included in our analysis: 1) Normal Muscle - Hofman - 121 - MAS5.0 - u133a: only the control group was selected (n=16) [1]; 2) Normal Muscle Skeletal - Asmann - 40 - MAS5.0 - u133p2: the analysis considered only young individuals to avoid age-related bias (n=20) [2]; 3) Normal Muscle - Gordon - 22 - MAS5.0 - u133p2: only the subset of young patients (n=14) was included (n=14) [3]; 4) Tumor Rhabdomyosarcoma - Davicioni - 147 - MAS5.0 - u133a: this dataset comprised 147 tumor samples (n=147) [4]; 5) Tumor Alveolar Rhabdomyosarcoma - Heiskanen - 186 - MAS5.0 - u133a: only patient tumor samples were used for the analysis, excluding cell line data (n=158) [4]; and 6) Tumor Rhabdomyosarcoma - Barr - 58 - MAS5.0 - u133p2 (n=58) [5].

*Cell lines and culture conditions*

All cell lines were cultured in MEM media (Biowest) supplemented with 10% fetal bovine serum (Sigma-Aldrich), 2mM L-glutamine, 1mM sodium pyruvate, 1x non-essential amino acids, 100U/ml penicillin, and 0.1mg/ml streptomycin (all from Biowest). Cells were maintained in a humidified incubator with 5% CO_2_ at 37°C.

*Lentiviral transduction*

To achieve TRIB3 knockdown, we employed shRNA technology using the pLKO.1 (constitutive) or pLKO-3xLacO (inducible by IPTG) vector systems from MISSION® shRNA (Sigma Aldrich). In the constitutive system, we tested five different shRNA sequences: TRCN0000197212 (referred to as sh12), TRCN0000295919 (sh19), TRCN0000196756 (sh56), TRCN0000199684 (sh84), and TRCN0000307989 (sh89). Among these, sh56 and sh84 were selected for further experimental procedures. Lentiviral particles were generated by co-transfecting the respective vectors with the packaging plasmids pMD2.G (RRID: Addgene_12259) and psPAX2 (RRID: Addgene_12260) in HEK293T cells (RRID: CVCL_0063). Subsequently, 2×10^5^ cells (RD, RH4, or RH30) were seeded in 60mm dishes, incubated overnight, and infected with the produced lentiviral particles. After 48 hours of infection, positive-transduced cells were selected by treating them with puromycin (1μg/ml, Sigma-Aldrich) for 72 hours. The knockdown efficiency was assessed by performing WB analysis.

*Cell survival*

The crystal violet assay was conducted to measure cell survival. Briefly, 5×10^2^ cells were seeded in 96-well plates. After 1, 3, 5, or 7 days of growth, cells were washed with PBS and stained with 0.5% crystal violet in 20% ethanol. Following three washes with PBS, the plates were air-dried overnight. The crystals were dissolved in 50 µL of a 15% acetic acid solution, and the absorbance was measured at 590nm using an Epoch Microplate Spectrophotometer (Biotek). Proliferation values were expressed as absorbance after subtracting the background OD590 using a blank control. Similarly, in the inducible model, after seeding the cells and an overnight incubation, IPTG was added at different concentrations, and proliferation was determined at 1, 3, 5, or 7 days after induction.

*Western blotting*

Protein expression was assessed through WB analysis. Total proteins were extracted using RIPA buffer supplemented with Halt™ Protease and Phosphatase Inhibitor Cocktail (Thermo Scientific). Approximately 20-30 μg of total protein was resolved in SDS-PAGE gels and transferred to a PVDF membrane. Following membrane blocking (with 5% non-fat milk or 5% BSA in TBST buffer), incubation with specific antibodies (Table S1) was performed. Secondary antibodies, including anti-mouse (Dako, P0260) and anti-rabbit (Sigma, A0545), were used. Chemiluminescence detection was carried out using Amersham™ ECL™ Prime Western Blotting Detection Reagent (GE Healthcare). The intensity of the bands was quantified using ImageJ software, as previously described [6]. The data were normalized to the β-Actin value.

| **Table S1**. Western blot antibodies conditions | | | | | | |
| --- | --- | --- | --- | --- | --- | --- |
| **Target protein** | **Cat No** | **Supplier** | **Source** | **Molecular Weight** | **1ry dilution** | **2ry dilution** |
| TRIB3 | ab75846 | Abcam | Rabbit | 40 kDa | 1/1,000 | 1/2,000 |
| Actin | sc-47778 | Santa Cruz | Mouse | 43 kDa | 1/20,000 | 1/20,000 |
| BAX | ABC11 | Millipore | Rabbit | 21 kDa | 1/2,000 | 1/4,000 |
| Capase-3 | ab32351 | Abcam | Rabbit | 32 kDa | 1/4,000 | 1/10,000 |
| Capase-3 cleaved | ab32351 | Abcam | Rabbit | 17 kDa | 1/2,000 | 1/4,000 |
| PARP | #9542 | Cell Signaling | Rabbit | 116 and 89 kDa | 1/2,000 | 1/4,000 |
| Phospho-AKT (Ser473) | #4060 | Cell Signaling | Rabbit | 60 kDa | 1/1,000 | 1/4,000 |
| AKT | sc-5298 | Santa Cruz | Mouse | 60 kDa | 1/1,000 | 1/5,000 |
| Phospho-PRAS40 (Tht246) | #2997 | Cell Signaling | Rabbit | 40 kDa | 1/1,000 | 1/8,000 |
| PRAS40 | #2691 | Cell Signaling | Rabbit | 40 kDa | 1/2,000 | 1/8,000 |
| Phospho-rpS6 (Ser235/236) | #4858 | Cell Signaling | Rabbit | 32 kDa | 1/5,000 | 1/8,000 |
| S6 | #2217 | Cell Signaling | Rabbit | 32 kDa | 1/2,000 | 1/8,000 |
| Phospho-FOXO1 (Ser322/S325) | ab60945 | abcam | Rabbit | 100 kDa (fusion protein) | 1/2,000 | 1/4,000 |
| FOXO1 | #2880 | Cell Signaling | Rabbit | 100 kDa (fusion protein) | 1/4,000 | 1/8,000 |
| MYCN | sc-53993 | Santa Cruz | Mouse | 67 kDa | 1/1,000 | 1/2,000 |
| Myogenin | sc-12732 | Santa Cruz | Mouse | 35 and 37 kDa | 1/1,000 | 1/4,000 |
| Lamin A/C | sc-376248 | Santa Cruz | Mouse | 69 and 62 kDa | 1/2,000 | 1/4,000 |
| GAPDH | sc-32233 | Santa Cruz | Mouse | 37 kDa | 1/5,000 | 1/5,000 |

*Co-immunoprecipitation*

For immunoprecipitation, cells were lysed in ice-cold RIPA buffer (25 mM Tris-HCl pH 7.6, 150 mM NaCl, 1% NP-40, 1% sodium deoxycholate, 0.1% SDS). Protein G Sepharose® 4 Fast Flow beads (Cytiva) bound to 1 µg of anti-TRIB3 (abcam, ab75846) or anti-AKT (Cell Signaling, #9272) were incubated with 500 µg of cell lysate for 2 hours at 4ºC with rotation. Then, immunoprecipitates were washed twice with RIPA buffer (for PAX3-FOXO1 inmunoprecipitation), and once with 50 mM Tris-HCl buffer. Proteins were eluted with Laemmli buffer, heated for 10 minutes at 75ºC and subsequently analyzed by WB.

*RNA isolation, retrotranscription, and quantitative real-time PCR*

Total RNA was isolated using a RNeasy Mini Kit (Qiagen) following the manufacturer’s instructions and quantified using a Nanodrop 2000 Spectrophotometer (Thermo Fisher Scientific). For retrotranscription, 1 μg of RNA was mixed with 1 μg of random primers (Thermo Fisher Scientific) in nuclease-free water and heated at 70 °C for 5 minutes. Then, 5 μL of 5x M-MLV reaction buffer (Promega), 5 μL of a mixture of 10 mM dNTPs, and 200U of M-MLV reverse-transcriptase (Promega) were added and maintained at 37 °C for 60 minutes. Real-time PCR was performed by mixing 0,5 μL cDNA with 5 μL 2X TaqMan Universal Master Mix (Thermo Fisher Scientific), 0,5 μL TaqMan assays (Thermo Fisher Scientific), and 4 μL nuclease-free water in PCR tubes. Then, each reaction mixture was transferred to MicroAmp 384-well plates (Thermo Fisher Scientific). PCR reaction was performed using an ABI PRISM 7900HT real-time PCR system (Thermo Fisher Scientific). A 40-cycle PCR was performed to detect TRIB3 (Hs01082394_m1, Thermo Fisher Scientific) and PAX3-FOXO1 (Hs03024825_m1, Thermo Fisher Scientific) expression. The housekeeping gene TBP (Hs00172424_m1, Thermo Fisher Scientific) was used as an endogenous control. Relative quantification of mRNA levels was performed by the 2^(-∆∆CT)^ method [7].

*Primary tumor mouse model*

A primary orthotopic tumor mouse model was established using the pLKO-3xLacO inducible model. SCID mice (Charles River Laboratories) were injected with 1×10^6^ RH30 cells into the right gastrocnemius muscle. The injected cells included those carrying the control plasmid (control group) or cells transfected with the plasmid containing sh84 (sh84 group). Tumor volume was calculated using the formula V=3/4π × ((length + width)/4)^3^, and tumor growth was monitored by measuring limb volume with a caliper. Once tumor growth was confirmed, the mice were randomized as follows: For the control group, mice were divided into two subgroups (non-induced (n=10) and induced (n=10)). In the sh84 group (n=32), the same randomization was applied, and 2 mice from each subgroup (non-induced and induced) were sacrificed at 7, 11, or 14 days (Figure S3a for scheme of the experimental design). Mice from the induced subgroups received drinking water containing 10 mM IPTG, with the water being replenished every 72 hours. Tumor volume was regularly measured, and animals were euthanized when the tumor volume reached 2000 mm^3^. Additionally, ethical endpoint criteria such as tumor size (>1 cm in diameter in any dimension), acute weight loss (>10% of total body weight), or poor general appearance of the animal were considered. All mice were housed under pathogen-free conditions. The experimental procedures were approved by the Ethics Committee of Animal Experimentation of the Vall d'Hebron Research Institute (CEEA 70/19) and were conducted in accordance with EU directive 2010/63/EU.

*Statistical analysis*

Experiments were conducted independently at least three times. The data are presented as the mean of each replicate ± standard deviation from three independent experiments. For the *in vivo* data, individual values of each animal are shown on the plots. Statistical analysis was performed after assessing normality and homogeneity of variance. Parametric or nonparametric tests were chosen accordingly. Comparisons between two groups were conducted using the t-test for parametric analysis or the Mann-Whitney U test for nonparametric analysis. Statistically significant differences among three or more groups were analyzed using one-way analysis of variance (ANOVA), two-way ANOVA, or Kruskal-Wallis test for non-parametric analysis, followed by post-hoc analysis. Additional details regarding the statistical analysis can be found in the respective sections. The level of significance was denoted by asterisks as follows: p < 0.05 (*), p < 0.01 (**), p < 0.001 (***), and p < 0.0001 (****). Plots and statistical analyses were performed using GraphPad Prism software (version 6.01).

**References**

1. Bakay M, Wang Z, Melcon G, Schiltz L, Xuan J, Zhao P, et al. Nuclear envelope dystrophies show a transcriptional fingerprint suggesting disruption of Rb-MyoD pathways in muscle regeneration. Brain. 2006.

2. Lanza IR, Short DK, Short KR, Raghavakaimal S, Basu R, Joyner MJ, et al. Endurance exercise as a countermeasure for aging. Diabetes. 2008.

3. Liu D, Sartor MA, Nader GA, Pistilli EE, Tanton L, Lilly C, et al. Microarray analysis reveals novel features of the muscle aging process in men and women. Journals Gerontol - Ser A Biol Sci Med Sci. 2013,

4. Davicioni E, Finckenstein FG, Shahbazian V, Buckley JD, Triche TJ, Anderson MJ. Identification of a PAX-FKHR gene expression signature that defines molecular classes and determines the prognosis of alveolar rhabdomyosarcomas. Cancer Res. 2006.

5. Sun W, Chatterjee B, Wang Y, Stevenson HS, Edelman DC, Meltzer PS, et al. Distinct methylation profiles characterize fusion-positive and fusion-negative rhabdomyosarcoma. Mod Pathol. 2015.

6. Gallo-Oller G, Ordoñez R, Dotor J. A new background subtraction method for Western blot densitometry band quantification through image analysis software. J. Immunol. Methods. 2018. p. 1–5.

7. Livak KJ, Schmittgen TD. Analysis of Relative Gene Expression Data Using Real-Time Quantitative PCR and the 2−ΔΔCT Method. Methods. 2001;25:402–8.

**ADDITIONAL FIGURES**


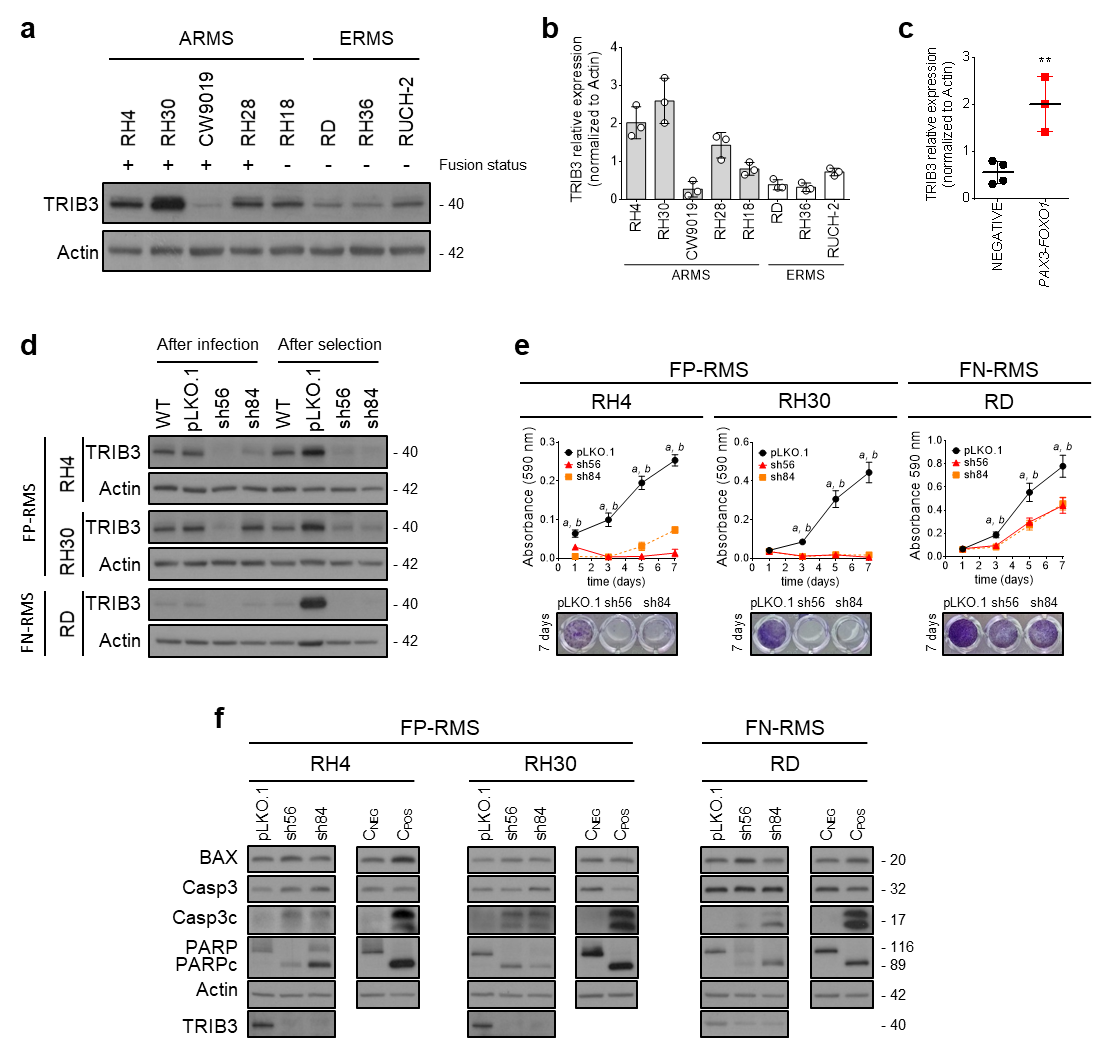


**Figure S1. TRIB3 is overexpressed in FP-RMS cell lines, and its genetic inhibition leads to impaired cell survival and increased apoptosis**. (a) TRIB3 protein expression was assessed by WB in RMS cell lines. The fusion status of each cell line is indicated by + for FP-RMS cell lines and - for FN-RMS. (b) Except for the CW9019 cell line, cell lines classified as ARMS exhibited the highest TRIB3 protein levels. (c) Fusion-positive cell lines demonstrated the highest TRIB3 protein levels. (d) To investigate the potential role of TRIB3, a constitutive model for genetic inhibition was optimized. sh56 and sh84 were selected to silence TRIB3 in RH4, RH30 (both FP-RMS), and RD (FN-RMS) cell lines. TRIB3 protein levels were determined 24 h after lentiviral infection and 72h after selection with puromycin. (e) Following constitutive TRIB3 knockdown, a clear impairment in cell proliferation was observed in FP-RMS cell lines compared to FN-RMS cell lines. (f) Analysis of apoptotic markers by WB confirmed the induction of apoptosis after TRIB3 silencing.

**
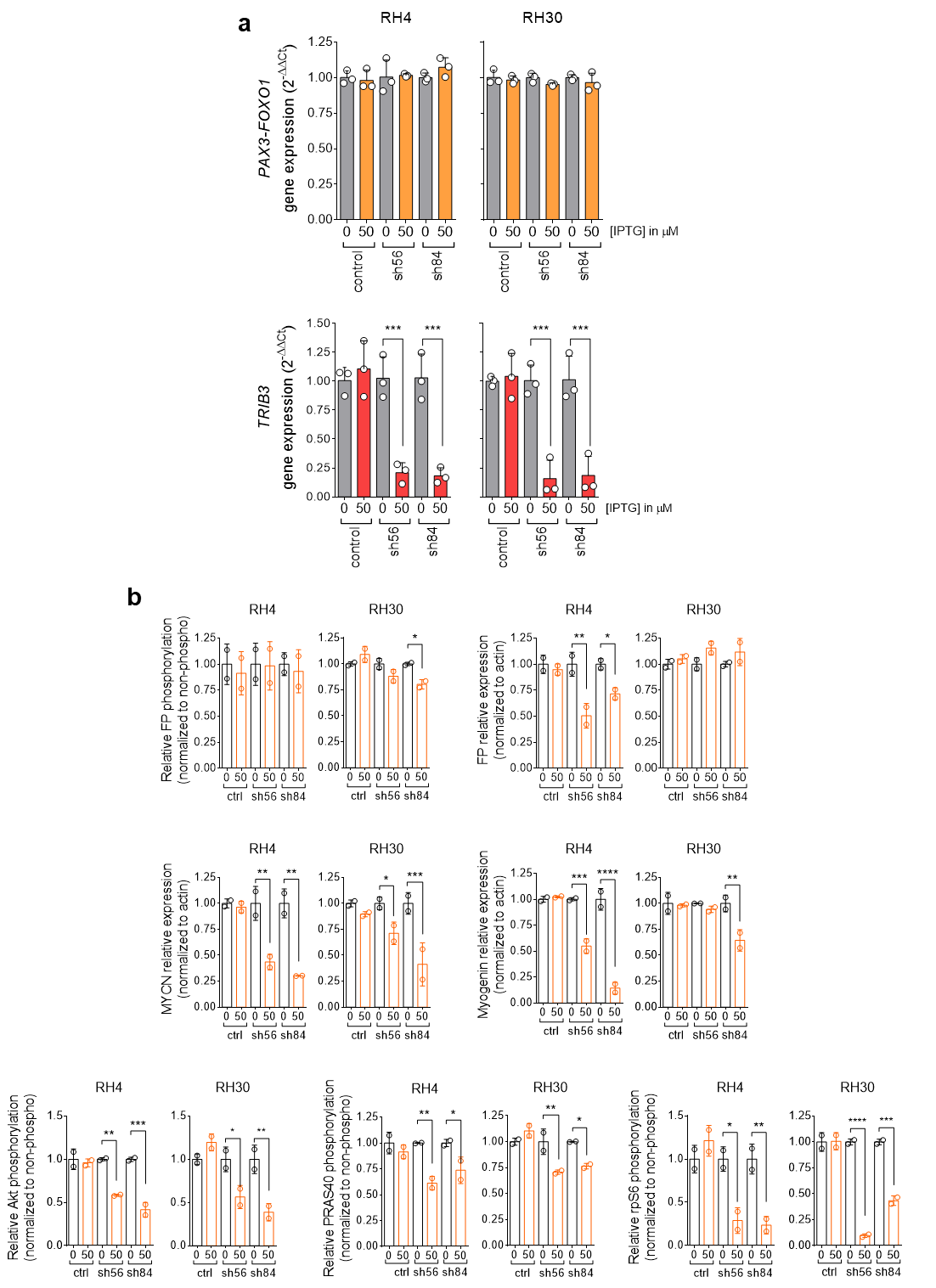
**

**Figure S2.** (a) Detection of relative gene expression levels (2^−ΔΔCT^) for the PAX3-FOXO1 fusion gene (upper panel) and TRIB3 (lower panel) in RH4 and RH30 cells in the presence or absence of IPTG. The expression values of the fusion protein remained unaltered even with the expression of shRNA against TRIB3. Upon induction by IPTG, the expression levels of the TRIB3 gene were significantly downregulated, confirming the validity of the inducible model. (b) Graphical representation of band intensity quantification of WB results corresponding to Figure 1e. Unpaired t test between control and induced in each condition was conducted.


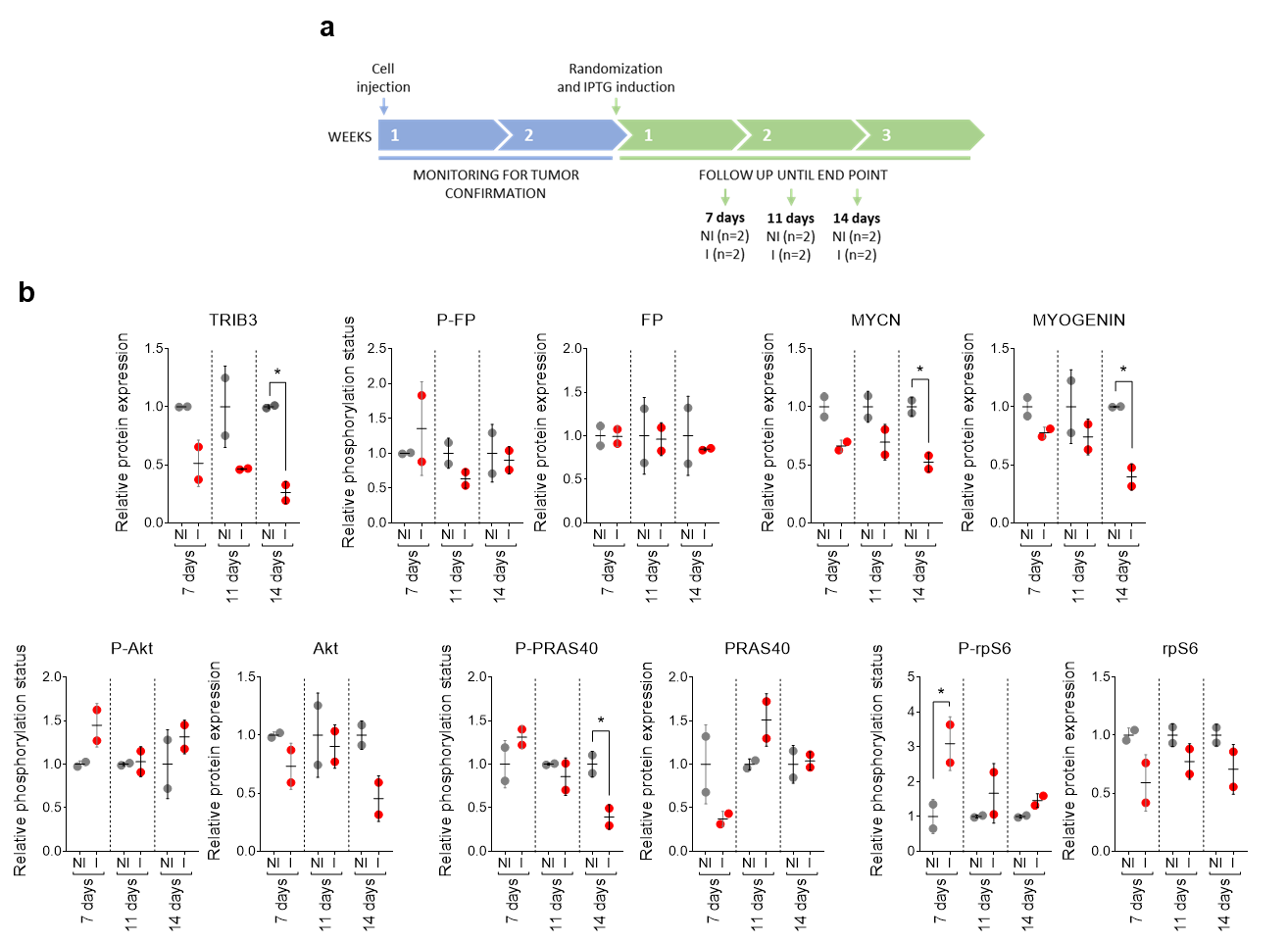


**Figure S3.** (a) Scheme of the in vivo experimental design. NI: non-induced. I: induced. (b) Band intensity quantification for mice samples sacrificed at 7, 11 and 14 days. Two-way ANOVA followed by Sidak's multiple comparisons test was conducted.
